# Integrative molecular analyses define correlates of high B7-H3 expression in metastatic castrate-resistant prostate cancer

**DOI:** 10.1038/s41698-022-00323-2

**Published:** 2022-11-02

**Authors:** Xiaolei Shi, Abderrahman Day, Hannah E. Bergom, Sydney Tape, Sylvan C. Baca, Zoi E. Sychev, Gabrianne Larson, Asha Bozicevich, Justin M. Drake, Nicholas Zorko, Jinhua Wang, Charles J. Ryan, Emmanuel S. Antonarakis, Justin Hwang

**Affiliations:** 1 Department of Medicine, University of Minnesota, Minneapolis, MN USA; 2 Division of Hematology, Oncology and Transplantation, University of Minnesota, Minneapolis, MN USA; 3 Dana-Farber Cancer Institute, Harvard Medical School, Boston, MA USA; 4 Department of Pharmacology, University of Minnesota, Minneapolis, MN USA; 5 Department of Urology, University of Minnesota, Minneapolis, MN USA; 6 Masonic Cancer Center, University of Minnesota, Minneapolis, MN USA; 7 Institute for Health Informatics, University of Minnesota, Minneapolis, MN USA; 8 Prostate Cancer Foundation, Santa Monica, CA USA

**Keywords:** Prostate cancer, Cancer genomics

## Abstract

B7-H3 (CD276) is an immune checkpoint overexpressed in prostate cancer with minimal expression in normal tissues and associated with poor prognosis, making it an excellent therapy target. We interrogated B7-H3 expression and its regulation in metastatic castration-resistant prostate cancer (mCRPC). We found greater expression of B7-H3 transcript relative to other immunotherapy targets (CTLA-4, PD-L1/2), including in tumors that lacked expression of prostate-specific membrane antigen (PSMA). Enzalutamide-resistant mCRPC cells demonstrated increased amounts of B7-H3, and this was associated with resistance signaling pathways. Using a machine-learning algorithm, the gene network of B7-H3 was strongly correlated with androgen receptor (AR) and AR co-factor (HOXB13, FOXA1) networks. In mCRPC samples, the B7-H3 promoter and distal enhancer regions exhibited enhanced transcriptional activity and were directly bound by AR and its co-factors. Altogether, our study characterizes molecular profiles and epigenetic regulation of B7-H3-expressing mCRPC tumors, which informs optimal precision-oncology approaches for mCRPC patients.

## Main

Localized prostate cancer (PC) is curable, but options are limited for recurrent or metastatic tumors developing resistance to androgen-deprivation therapy (ADT) or AR targeted therapy (ART), known as metastatic castration-resistant prostate cancer (mCRPC). Overexpressed tumor antigens, such as PSMA, are targets of novel PET imaging approaches^1^ as well as precision therapeutics (^177^Lu-PSMA-617) in mCRPC (https://www.accessdata.fda.gov/drugsatfda_docs/label/2022/215833s000lbl.pdf). Identifying additional mCRPC tumor antigens contributes to new strategies to develop precision antibody-drug conjugates that permit immuno- or cellular therapies^2,3^.

B7-H3 is a transmembrane glycoprotein in the B7 immune checkpoint superfamily^4^. Other well-known members, such as PD-L1 and CTLA-4, are targets in various hematologic and solid tumors^5–7^. B7-H3 is overexpressed in several cancers including prostate cancer, with minimal expression in normal prostatic tissue^8–12^. Higher expression of B7-H3 correlates with poor cancer prognosis^8^. B7-H3 has implications for cancer cell transformation and metastasis, and is thought to have a significant effect on the tumor microenvironment and immune suppression^11,13^. New strategies have been developed to target B7-H3 through antibody-dependent cell-mediated cytotoxicity^14^, antibody**-**drug conjugates^15^ and linking with immunotherapy such as chimeric antigen receptor-T cell^16^ or NK cell therapies^17^. However, little is known about the molecular features and regulatory mechanisms of B7-H3 in mCRPC, which prevents the optimal design of such targeted interventions and precludes rational patient selection. To address these barriers, we characterized the genomic, transcriptomic and epigenomic features of B7-H3 expression in mCRPC.

**Fig. 1:**
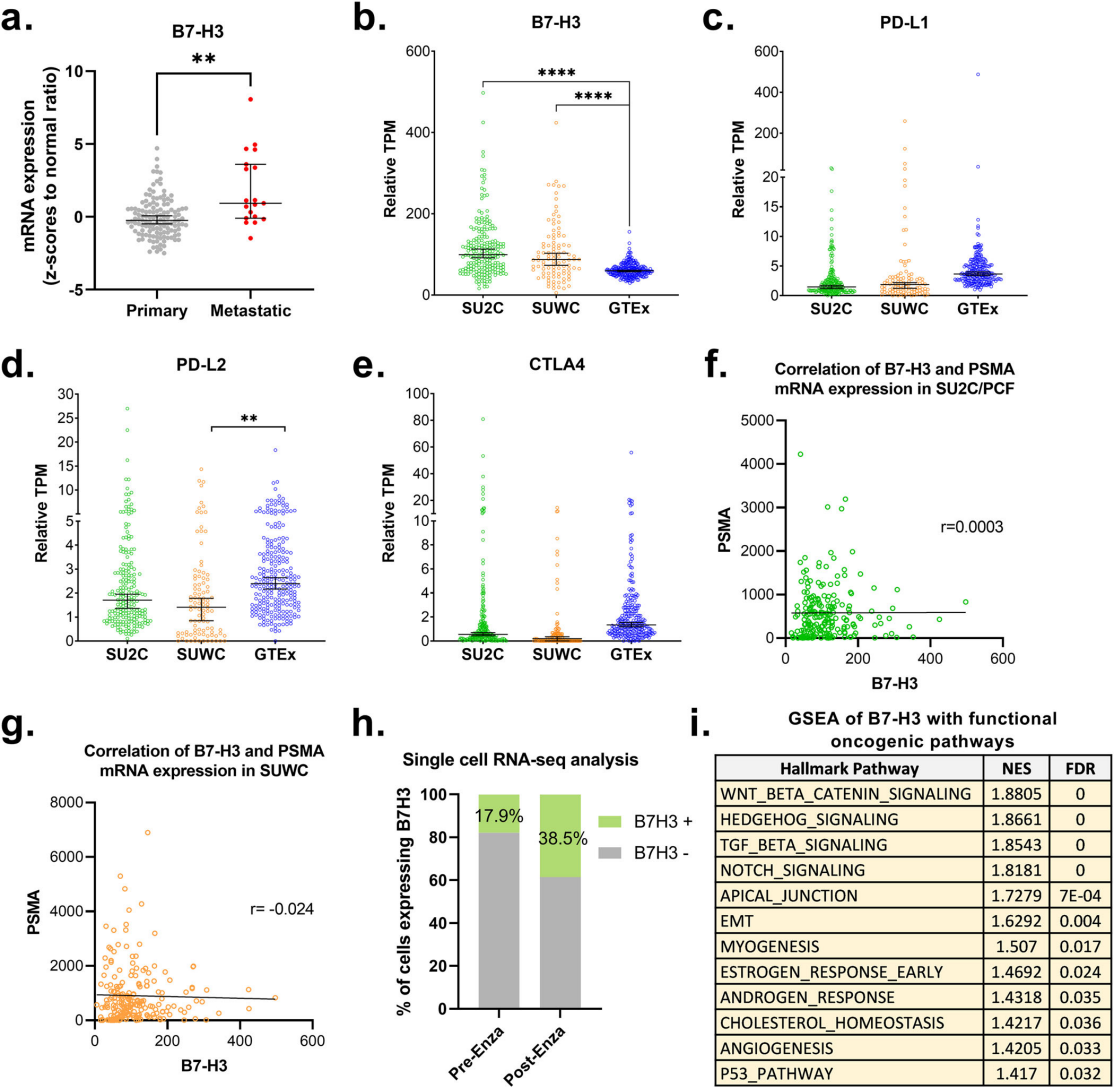
B7-H3 is selectively overexpressed in mCRPC. **a** mRNA expression of B7-H3 in primary and metastatic prostate cancer (PC). Whole-transcriptome sequencing (WTS) data were obtained and analyzed from MSKCC 2010 (primary PC in gray, *n* = 131; metastatic PC in red, *n* = 19). All data are median with 95% CI. Statistical significance was using student *t*-test. **P < 0.01. **b**–**e** mRNA expression of B7-H3, PD-L1, PD-L2, and CTLA-4, respectively, in mCRPC (SU2C, green; SUWC, orange) and normal prostate tissue (GTEX, blue). Whole-transcriptome sequencing (WTS) data were obtained and analyzed from SU2C/PCF (*n* = 208, mCRPC), SUWC (*n* = 101, mCRPC), and GTEx (*n* = 245, benign prostate tissue). Data of other B7 family genes are shown in Supplementary Fig. 2. All data are median with 95% CI. Statistical significance was assessed using one-way ANOVA for multiple comparisons. ****P < 0.0001. **f**, **g** Lack of correlation of mRNA expression of B7-H3 and PSMA in mCRPC datasets SU2C/PCF (*n* = 208, r = 0.0003) and SUWC (*n* = 101, r = −0.024), respectively. Associations were determined by Pearson correlations. **h** Percentage of cells expressing B7-H3 (B7-H3 positive, green; B7-H3 negative, gray) before (17.9%) and after (38.5%) enzalutamide treatment. ScRNA-Seq analysis was performed on cells from patient before (*n* = 112) and after enzalutamide (*n* = 83) treatment. **i** GSEA of B7-H3 with functional oncogenic pathways. WTS data of mCRPC datasets SU2C/PCF (*n* = 208) and SUWC (*n* = 101) mCRPC were combined for GSEA. NES cutoff value 1.4. NES normalized enrichment score. FDR false discovery rate.

We first evaluated transcript expression profiles of B7-H3 (CD276) and other immune-regulatory genes in mCRPC. We conducted bioinformatic interrogations on whole-exome (WES) and whole-transcriptome sequencing (WTS) data from the datasets including MSKCC 2010 (*n* = 131, primary PC; *n* = 19, CRPC)^18^, SU2C/PCF (*n* = 208, mCRPC)^19^, SUWC (*n* = 101, mCRPC)^20^, and GTEx (*n* = 245, benign prostate tissue) datasets^21^. The MSKCC samples included both primary and metastatic tumors processed through the same platform. mRNA expression of B7-H3 was significantly increased in metastatic PC compared to primary PC (*p* = 0.004) (Fig. 1a). In other mCRPC datasets, expression of B7-H3 was significantly elevated in both mCRPC datasets compared to benign prostate tissues (median TPM 115, 87 vs. 60, *p* < 0.0001) (Fig. 1b). We also evaluated the association of the expression of B7-H3 mRNA with protein levels and found significant association in 369 cancer cell lines (*p* = 1.03E-72) (Supplementary Fig. 1a). In 10 patient-derived xenograft (PDX) models of castration-resistant prostate cancer (LuCaP PDX series)^22^, we detected B7-H3 expression in each PDX tumor pair and found a positive trend between mRNA and protein expression (*n* = 10, r = 0.52, *p* = 0.06) (Supplementary Figs. 1b–c and 4). Based on TPM, other B7 family members including PD-L1 (CD274), PD-L2 (PDCD1LG2), and CTLA-4, exhibited reduced expression in both mCRPC datasets and had limited overall transcript abundance compared to B7-H3 (Fig. 1c–e). Other immunological markers exhibited low abundance or were not overexpressed in mCRPC (Supplementary Fig. 2). The expression of B7-H3 in mCRPC was independent of that of PSMA, and exhibited robust expression even in PSMA-low tumors (Fig. 1f–g). This suggests that targeting B7-H3 could be an attractive alternative for PSMA-negative/low mCRPC patients.

To examine how mCRPC tumor cells regulate B7-H3 expression in response to the ART, enzalutamide, we analyzed single-cell mRNA sequencing (scRNA-seq) data of paired biopsy samples from one patient (pre- and post-enzalutamide). We found an increased proportion of B7-H3-expressing tumor cells post-enzalutamide (38.5%) relative to pre-enzalutamide (17.9%) (Fig. 1h). Based on the genomic alterations in mCRPC with high B7-H3 expression, B7-H3 was associated with several known resistance markers including PTEN inactivation and AR-V7 detection^19,20,23^ (Supplementary Fig. 3). We conducted Gene Set Enrichment Analysis (GSEA)^24^ on mCRPC datasets (SU2C/PCF, *n* = 208 and SUWC, *n* = 101) and identified B7-H3 was enriched of TGF-beta, WNT, and Epithelial-to-Mesenchymal Transition (EMT) signaling pathways (Fig. 1i); each has been associated with resistance to enzalutamide^25–27^. Altogether, we found robust B7-H3 expression in mCRPC patients with existing molecular or signaling features that promote resistance to ADT and/or ART.

**Fig. 2:**
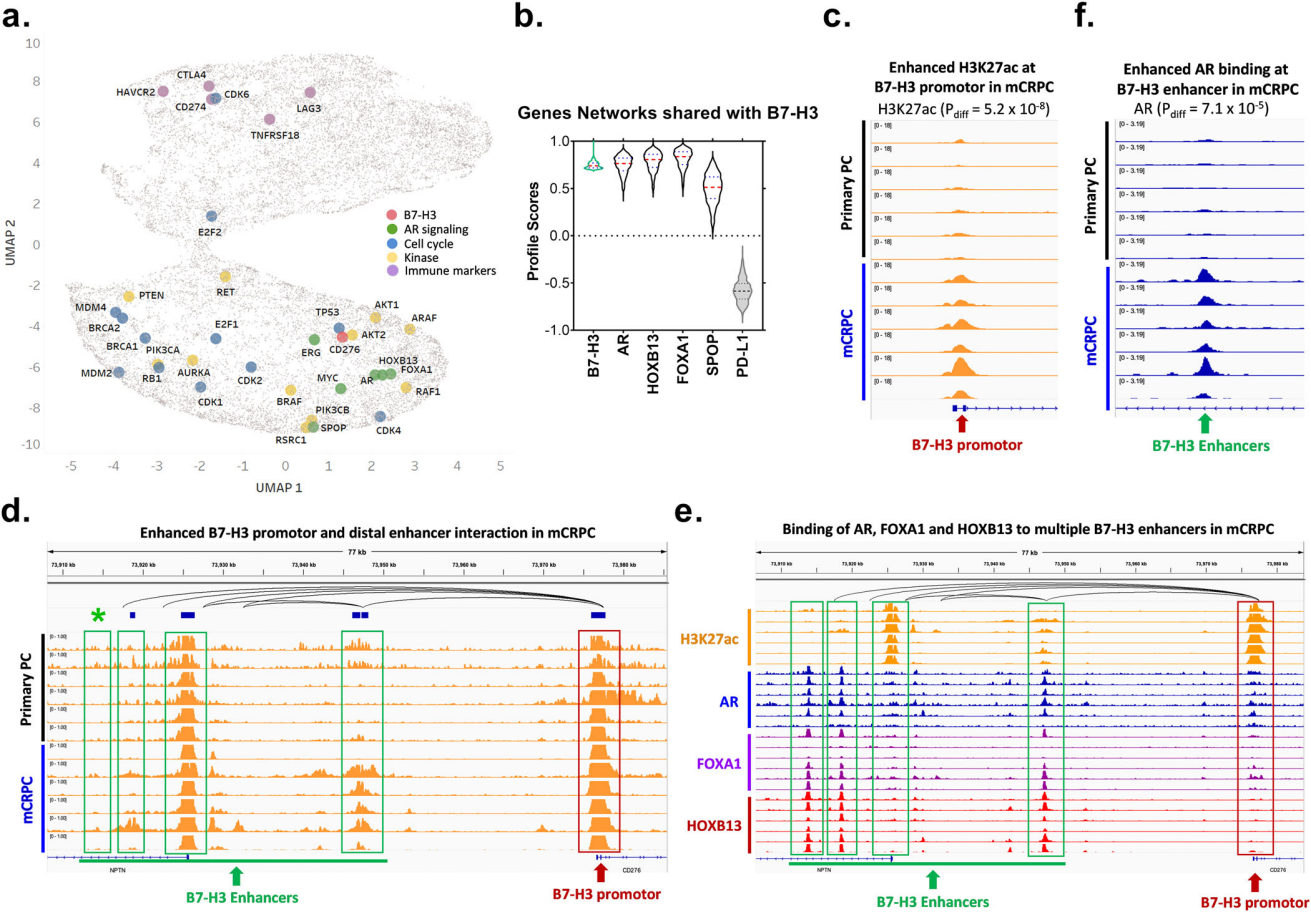
B7-H3 is significantly associated with and regulated by AR-signaling pathways. **a** Machine-learning (ML)-based UMAP analysis of the association between B7-H3 and key PC pathways. Each dot in UMAP represents one gene. The spatial distance between two genes represents the similarity of their gene networks. Key PC pathways are visualized including AR signaling (green), Cell cycle (blue), Kinases (yellow), and Immune markers (purple) along with B7-H3 (pink). **b** ML-based analysis of the gene-network association between B7-H3 and key AR-signaling pathway genes in mCRPC patients (SU2C/PCF, *n* = 208). Data are shown in violin plots, in which red lines represent median and blue lines represent first quartile (lower) and third quartile (upper). The boundary of the violin represents the range of all data points. The degree of overlap of the plots represents the similarity of the networks they are associated with. PD-L1 was used as a negative control. **c** Comparison of H3K27ac enhancement at B7-H3 promoter in mCRPC and primary PC from representative patient-derived xenografts (PDXs)^22^. Pdiff indicates the FDR-adjusted *P*-value for comparison between primary prostate cancer and mCRPC using DESeq2. **d** Enhanced interaction between B7-H3 distal enhancers (green box) and its promoter (red box) in mCRPC as compared to primary PC. Top track indicates H3K27ac HiChIP data from LNCaP, which reflects long range chromosomal interactions in LNCaP cells. **e** Binding of AR, FOXA1, and HOXB13 to multiple putative B7-H3 enhancer sequences (green box) in mCRPC. **f** Increased AR binding in mCRPC at one of the putative *CD276* enhancers indicated in **d**, labeled with green *. Six representative AR ChIP-seq profiles of primary prostate cancer and mCRPC are shown^32^.

To enhance our mechanistic understanding of B7-H3 expression in mCRPC, we developed a machine-learning algorithm that quantitatively measures the degree of all gene-to-gene interactions to construct gene networks for all detectable genes. We used this algorithm to compare the degree of gene-network interactions between B7-H3 and all other detectable gene networks in the 208 mCRPC patients from the SU2C/PCF study. The overarching degree of gene-network association was visualized on UMAP, depicted through distances on an x-y plane. Remarkably, B7-H3 networks were closely clustered with those of AR, as well as with FOXA1, HOXB13, SPOP, MYC, and ERG (Fig. 2a). CTLA-4, PD-L1, PD-L2, and other immune markers were in distinct clusters (Fig. 2a), which indicated a lack of association with AR-signaling genes. We further examined the similarities of gene networks of B7-H3 and key regulators of AR signaling on a violin plot, in which the degree of overlap represents similarity. We observed that the B7-H3 gene network overlapped with those of AR, HOXB13, and FOXA1, and to a lesser degree with SPOP, but exhibited no intersection with PD-L1 (Fig. 2b). Altogether, these analyses suggest a robust convergence between B7-H3 and multiple genes with known functions in AR signaling.

B7-H3 is regulated epigenetically in nasopharyngeal carcinoma^28^ and glioblastoma^29^, via histone acetylation and DNA methylation at the promotor, respectively. The convergence between B7-H3 and AR signaling that we identified agreed with prior studies^30^. We thus sought to interrogate this mechanism of regulation through CHIP-seq data from both primary prostate cancer and mCRPC xenograft samples^31,32^. Remarkably, we observed enhanced histone-3-lysine-27 acetylation (H3K27ac) marks at the B7-H3 promoter and at putative B7-H3 distal enhancers in mCRPC as compared to primary prostate cancer (Fig. 2c, d), which reflected molecular mechanisms that increased transcription of B7-H3 in mCRPC. Further, we found that AR (and its co-regulators HOXB13 and FOXA1) were directly bound to B7-H3 enhancers (Fig. 2e). Notably, we found that AR exhibited selective binding to one of the B7-H3 putative enhancers in mCRPC as opposed to primary tumors (Fig. 2f). Although AR signaling is active at all stages of prostate cancer, our ChIP-seq analysis illustrated differential epigenetic regulation of B7-H3 transcripts in mCRPC compared to primary prostate cancer.

Our findings provide support that B7-H3-targeting therapies can fulfill an unmet medical need for ADT/ART-resistant mCRPC patients. Strategies to target B7-H3 with checkpoint inhibitors (NCT03729596), monoclonal antibodies (NCT02923180), antibody-drug conjugates (NCT03729596, NCT04145622), or tri-specific killer engager (TriKE) agents, are currently under investigation^33,34^. These therapeutics could be rationally designed for mCRPC patients that harbor ADT/ART-resistant biomarkers (e.g., PTEN loss, AR-V7 or ERG fusion) or other oncogenic signaling pathways (WNT, EMT, TGF-Beta). Further, targeting B7-H3 may be relevant in mCRPC patients with limited expression of PSMA, although our analysis did not address the status of B7-H3 in neuroendocrine/ small-cell prostate cancers. Our findings established a mechanistic connection between B7-H3 expression and AR-related signaling in mCRPC. This may also hold true in high-risk localized prostate tumors, since B7-H3 immunostaining is reduced after intense neoadjuvant ADT given before radical prostatectomy^30^. Finally, the epigenetic modifications we found may act as surrogates to measure B7-H3 levels from noninvasive liquid biopsies that include circulating-tumor DNA from mCRPC patients^27^.

## Methods

### Gene expression analysis

We normalized the expression of all commonly detected genes across four clinical cohorts (GTEx^21^, TCGA prostate cancer, SU2C/PCF^19^, SUWC^20^). These were originally mapped with distinct platforms and expression units. For each dataset, expression measurements were first converted into transcripts per million (TPM). The SU2C 2019 dataset was converted from fragments per kilobase of exon per million mapped fragments (FPKM) to TPM by scaling each sample such that the sum of expression came to 1 million for each sample^35^. The TCGA dataset was converted from RNA-seq by Expectation Maximization (RSEM) estimated transcript fraction to TPM by multiplying each value by 1 million^36^. The GTEx and SUWC datasets were already in TPM units. The TPM expression data for the total samples (*n* = 1102) in each dataset was merged by gene symbols that were common in all datasets. This merged dataset included 17,044 genes based on scaling each sample by a constant such that the total expression values in each sample sums to 1 million to generate pseudo-TPM values. The final matrix was then used to compare or correlate normalized expression of B7-H3 and all test genes in prostate tissue or metastatic prostate cancer samples. Statistical significance was determined by one-way ANOVA and correlation coefficients based on *p*-values < 0.05. The scripts and normalized data used to generate the data are shared at: git@github.com:bergo015/GeneNetworkingB7H3.git.

### Protein and RNA expression analysis

To analyze the correlation between RNA and protein levels of B7-H3 (CD276), we utilized the Data Explorer tool from the DepMap Portal (https://depmap.org/portal/interactive/)^37^. On this portal, we elected to examine the public RNA (22Q2) and Proteomics data. This interface generated Pearson or Spearman correlations on CD276 expression levels and provided the statistical significance based on the linear regression. We exported the data points to generate Supplemental Fig. 1a.

### Single-cell RNA sequencing analysis

The study from He et al.^25^ included one patient with paired biopsies prior to enzalutamide treatment and post resistance. Samples were collected with informed consent and ethics approval by the Dana-Farber/Harvard Cancer Center Institutional Review Board under protocol nos. 09–171, 11–104, 13–301, and 01–045. In these paired samples, we examined the tumor cells based on established mechanism to infer tumor cells^25^. This yielded 112 cells prior to enzalutamide treatment and 83 cells post resistance. To analyze these cells, we determined the positivity of B7-H3 expression based on absolute count values. We assigned B7-H3-positive or -negative cells based on the detection of B7-H3 transcripts in each tumor cells. We determined the proportion of cells with B7-H3 expression by dividing the number of cells expressing B7-H3 by the total cell numbers before enzalutamide treatment and post resistance.

### Gene-network construction and analyses

We devised an algorithm that computes gene networks based on serial correlations that measure relative association between all gene pairs. Here we utilized whole-transcriptome sequencing data based on SU2C/PCF^19^. The input and computing were performed on the servers at the Minnesota Supercomputing Institute. This yielded the gene networks for all 19,135 genes in the format of a square matrix with the rows and columns representing gene IDs. Each element in the 19,135 × 19,135 matrix represents a similarity score of the two genes and their networks. The degree of similarity is measured by numerical values between −1 (highly discordant networks) to 1 (highly similar networks). To generate a B7-H3 gene-network signature, we included all gene networks based on a similarity score of ≥0.7 in reference to B7-H3. We then compared how all elements in this B7-H3 gene-network signature were associated with all other test genes. The degree of similarity to B7-H3 is assessed by the degree of overlap of the B7-H3 gene-network signature and all test genes in a violin plot. To visualize the similarity of the B7-H3 gene network in reference to all other genes, we reduced the 19,135 × 19,135 output matrix using Uniform Manifold Approximation and Projection (UMAP) to 2 dimensions (19,135 × 2 matrix). Plotting the two dimensions on a scatter plot, each point represents a gene, and the distance between each gene and B7-H3 represents the relative gene-network similarity. The scripts, input, and resulting data are provided at git@github.com:bergo015/GeneNetworkingB7H3.git.

### Gene set enrichment analysis (GSEA)

Gene profiles were generated by associating B7-H3 expression to all other detected genes in all samples. The mRNA expression profiles were obtained by averaging the mRNA expression of each gene based on whole-transcriptome sequencing data from datasets SU2C/PCF (*n* = 208, mCRPC) and SUWC (*n* = 101, mCRPC)^19,20^. GSEA^24^ pre-ranked analyses was then performed on this profile to identify enrichment of gene signatures from the 189 C6 oncogenic signatures in MSigDB^38^. Based on the output files, we tabulated the normalized enrichment scores that were significant based on False Discovery Rates (FDR).

### ChIP-seq analysis

ChIP-seq data were previously reported^32^ and are available under GEO accession number GSE130408. Primary tumors were obtained from prostatectomy specimens; mCRPC samples were obtained from LuCaP series PDXs^22^. Differential ChIP-seq analysis of AR binding and H3K27ac were performed in Pomerantz et al.^32^ using DESeq2 and included 23 primary cancer and 15 mCRPC-derived PDXs. The epigenetic regulation at specific chromosomal coordinates was visualized in each figure using IGV viewer.

### Immunoblots

Immunoblots was performed following the Licor-system western blot detection protocol (licor.com/bio/support). 20ug of whole cell lysate from LNCaP and HL60 cells were ran in 4–20% SDS-page gel (Biorad, Cat# 4568094) and transferred to Immobilon-FL Transfer Membrane PVDF 0.45 uM pore size (Immobilon cat# IPFL00010). Protein was probed with antibodies against B7-H3 (Invitrogen, Cat # MAS-15693; R&D Systems, Cat # AF1027; Bethyl Laboratories, Cat # A700-026) and GAPDH (Santa Cruz, Cat # sc-32233). Precision plus was used as the protein standard (Bio-Rad, Cat# 161-0363). Primary antibodies were diluted 1:500 and incubated in 5% BSA, PBX and 0.2% tween-20 blocking solution. Secondary antibodies (IRDye 800 CW Goat-anti Mouse, Licor, Cat # 925-32210; IRDye 800 CW Goat-anti Rabbit, Licor, Cat # 925-32211; HRP-Conjugated Mouse-anti Goat, Santa Cruz, Cat # SC2354) were diluted 1:2000 and incubated in 5% BSA, 1× PBX, and 0.2% tween-20 and 0.01% SDS. Bio-rad Chemi-Doc MP Imaging system was used to scan the PVDF membrane for HRP activity detection. The protein expression of B7-H3 was normalized to GAPDH (loading control). All blots were derived from the same experiment and they were processed in parallel. The original scans of the blots are shown in Supplemental Fig. 4.

### Statistics

The statistical significance of the mRNA expression of B7-H3 among the three datasets was assessed using one-way ANOVA. The associations between B7-H3 and PSMA were determined through Pearson correlations. The statistical significance was assessed using Chi-square and Fisher’s exact tests for categorical comparison between B7-H3 and genetic alterations. Student *t*-test was applied for AR-V7 and mRNA expression comparison. Graphs were generated using Prism Graphpad.

### Reporting summary

Further information on research design is available in the Nature Research Reporting Summary linked to this article.

## Supplementary information

Supplemental figure 1, 2, 3, 4

REPORTING SUMMARY

## Data Availability

Datasets derived from public resources and these resources are provided within the article and below. (1) The MSKCC 2010^18^ data are deposited in at NCBI GEO under accession GSE21032. The analyzed data can also be accessed and explored through the MSKCC Prostate Cancer Genomics Data Portal: http://cbio.mskcc.org/prostate-portal/#x201D. 10.1016/j.ccr.2010.05.026 (2010). (2) The SU2C/PCF^19^ data are available in Dataset S1 and at www.cbiportal.org, and have been deposited in GitHub, https://github.com/cBioPortal/datahub/tree/master/public/prad_su2c_2019. 10.1073/pnas.1902651116 (2019). (3) For the SUWC dataset^20^, the accession number for the raw sequencing data reported in the paper is dbGAP: phs001648.v1.p1. 10.1016/j.cell.2018.06.039 (2018). (4) The GTEx^21^ data are available through the GTEx portal (www.gtexportal.org). 10.3390/jpm5010022 (2015). (5) For the single-cell sequencing (scRNA-seq) dataset^25^, scRNA-seq expression and clustering data generated in this study are available at https://singlecell.broadinstitute.org/single_cell/study/SCP1244/transcriptional-mediators-of-treatment-resistance-in-lethal-prostate-cancer. Raw sequence data generated in this study are being deposited in dbGaP (accession phs001988.v1.p1). 10.1038/s41591-021-01244-6 (2021). (6) The proteomics data of the 369 cell lines used in Supplementary Fig. 1a were obtained from DepMap Portal using the Data Explorer feature. https://depmap.org/portal/interactive/.

## Code availability

The basic machine-learning operation source code, input datasets, and output matrices are provided in the following link: git@github.com:bergo015/GeneNetworkingB7H3.git
