# Supplementary material for: Integrative molecular analyses define correlates of high B7-H3 expression in metastatic castrate-resistant prostate cancer
Source: NPJ Precis Oncol. 2022 Nov 2;6:80. doi: 10.1038/s41698-022-00323-2 (PMC9630314; doi:10.1038/s41698-022-00323-2)
Supplement: Supplementary file 1 — Supplemental figure 1, 2, 3, 4 [file 41698_2022_323_MOESM1_ESM.pdf]

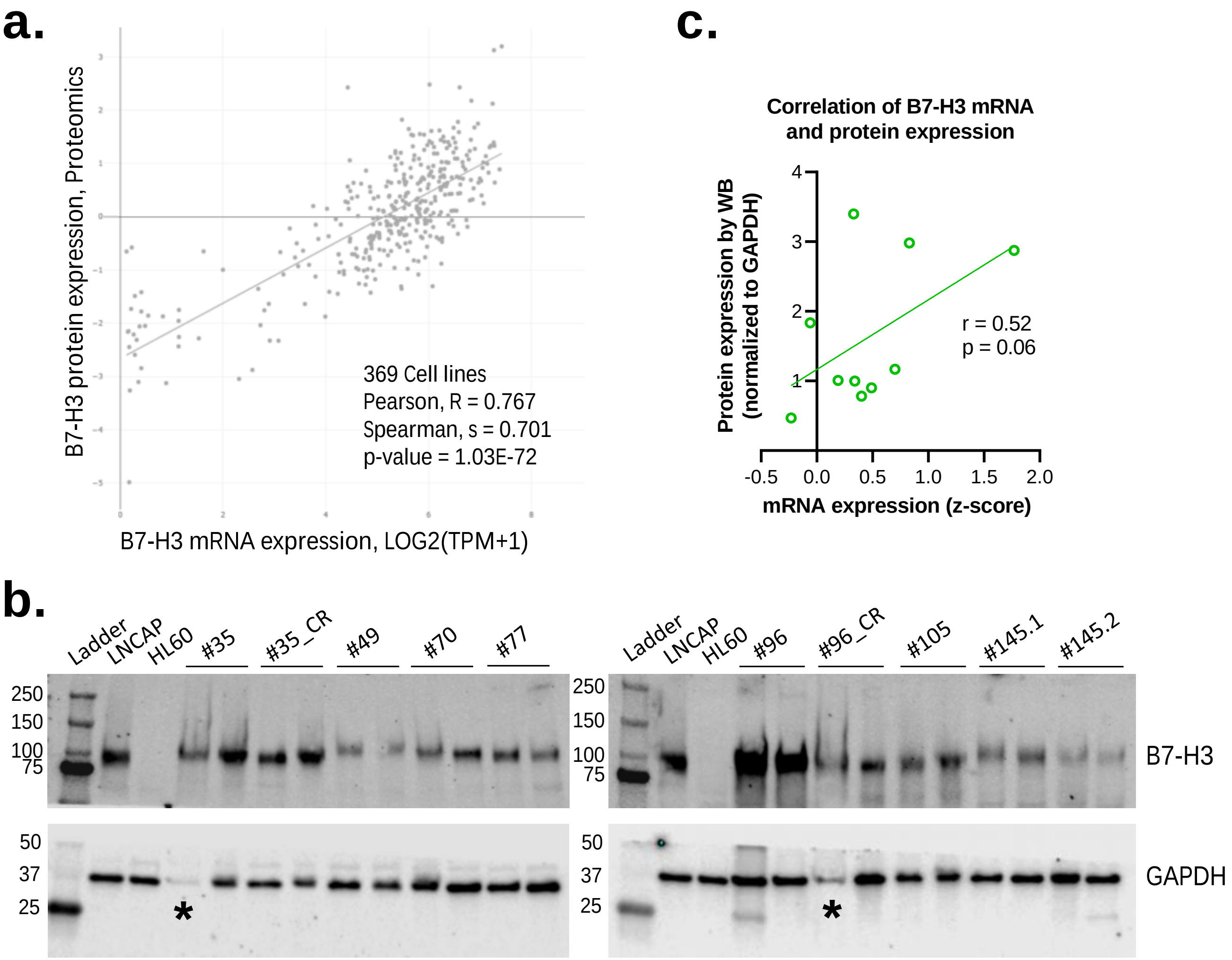

Supplementary Figure 1. Comparison of B7-H3 mRNA and protein expression. (a) Correlation of B7-H3 mRNA and protein expression in cell lines. 369 cancer cell lines from over 35 lineages were examined based on the Cancer Cell line Encyclopedia. The expression of B7-H3 (CD276) was based on RNA-seq and proteomics data. (b) A representative image of quantitative Western immunoblot (WB) analysis of B7-H3 in 10 LuCap PDX series. LNCaP, positive control; HL-60s, negative control; GAPDH, loading control. Each LuCap PDX tumor is duplicated. (c) Correlation of B7-H3 mRNA and protein expression in 10 LuCap PDX series. The protein expression of B7-H3 was normalized to that of GAPDH. The average B7-H3 protein/GAPDH ratio of the two samples for each LuCaP PDX tumor was used to generate Figure S1c. Association was determined by Pearson correlations. One sample from #35 and #96_CR (labeled with *), respectively, were excluded due to minimal GAPDH expression concerning for sample lysis.

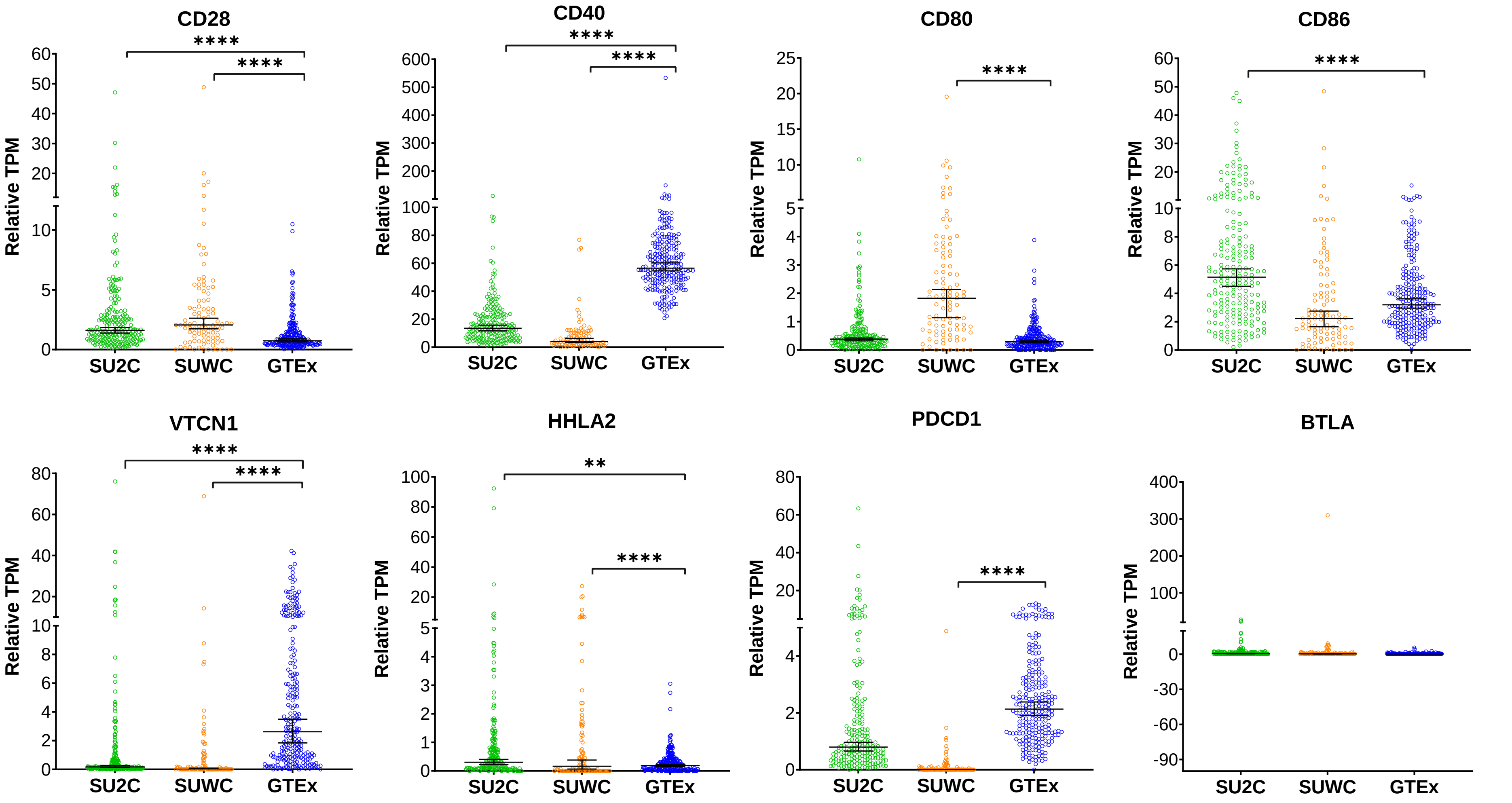

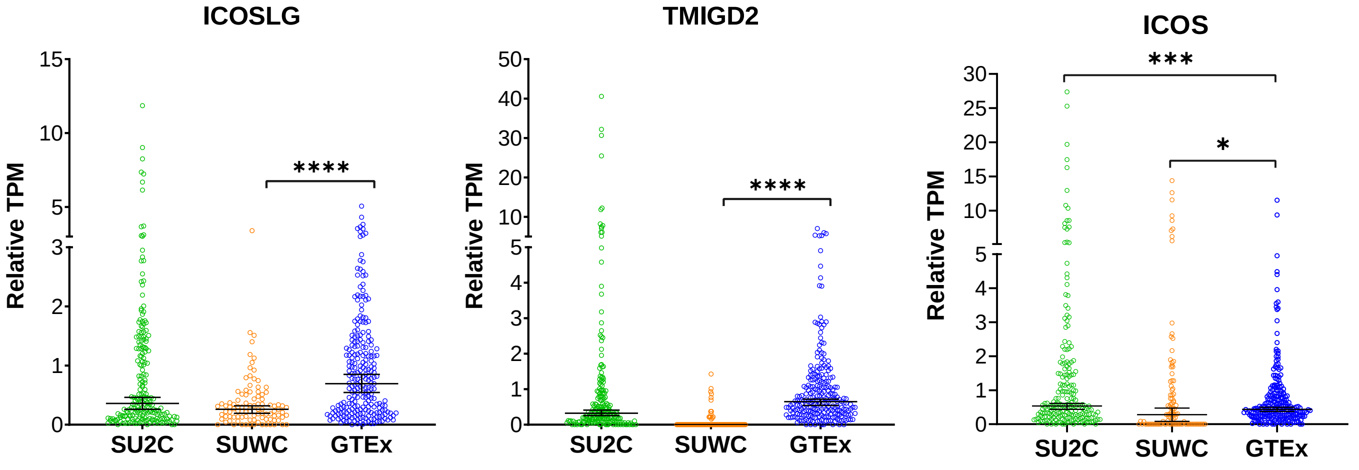

Supplementary Figure 2.
mRNA expression of B7 superfamily genes in mCRPC and normal prostate tissues. WTS data was obtained and analyzed from SU2C/PCF (n = 208, mCRPC), SUWC (n = 101, mCRPC), and GTEx (n = 245, benign prostate tissue). All data are median with 95% CI. Statistical significance was assessed using one-way ANOVA for multiple comparisons. * $P < 0.05$, ** $P < 0.01$, *** $P < 0.001$, **** $P < 0.0001$.

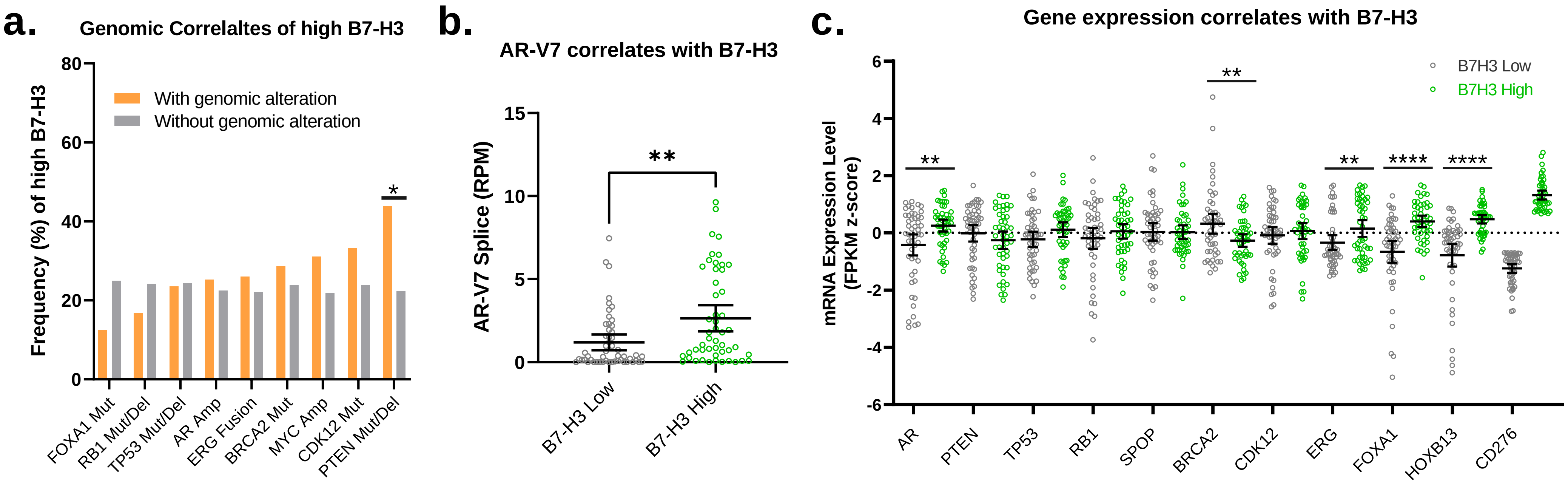

Supplementary Figure 3. B7-H3 high mCRPC is associated with certain genomic and transcriptional features in mCRPC. (a) Frequency of genomic alterations in mCRPC patients with high B7H3 expression. (b) AR-V7 variants and (c) mRNA expression of key mCRPC genes in high and low B7-H3-expressing mCRPC. WTS, copy number variation, structural variant, and mutation data were obtained and analyzed from mCRPC dataset SU2C/PCF (n = 208). High B7-H3 group was defined as the top 25% of B7H3 expressing samples and low B7H3 group was defined as the bottom 25% of B7H3 expressing samples. Only one sample from each patient was counted and patients with samples within both high and low B7H3 groups were excluded from the analysis. A total of 48 samples in B7H3 high group and 50 samples in B7H3 low group were included in the analysis. Statistical significance was assessed using Chi-square and Fisher's exact tests for categorical comparison between B7-H3 and genetic alterations. Student *t*-test was applied for AR-V7 and mRNA expression comparison. All data are mean with 95% CI. * $P < 0.05$, ** $P < 0.01$, **** $P < 0.0001$. Amp, amplification. Del, deletion. Mut, mutation.

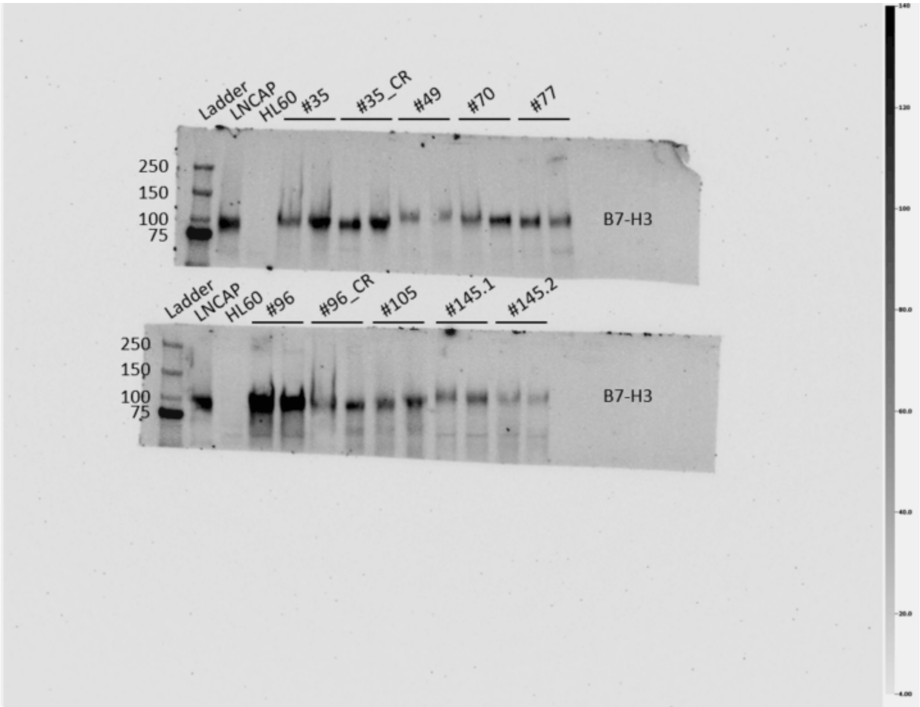

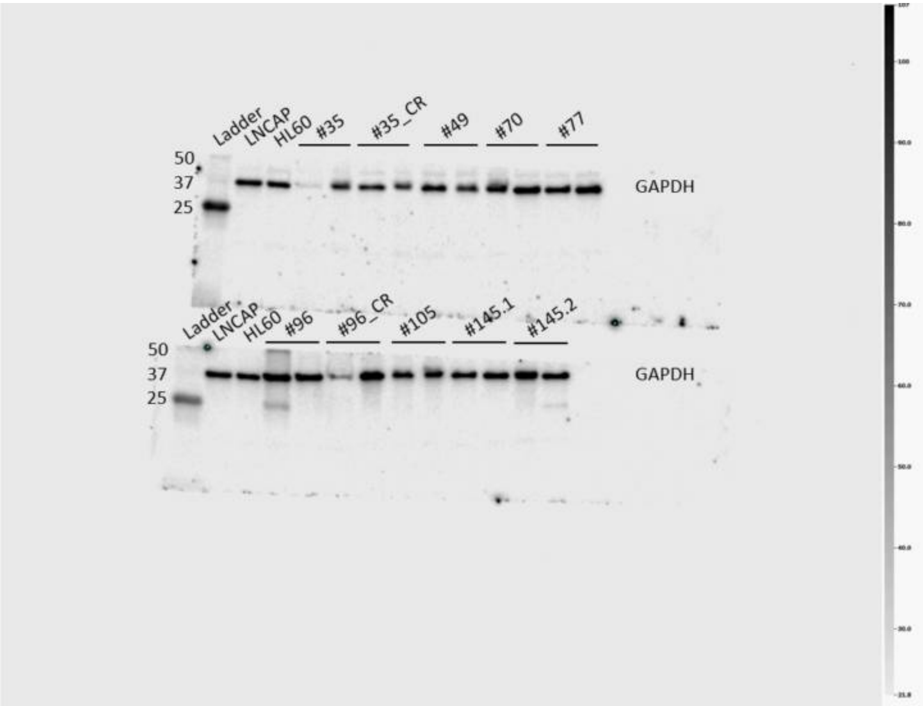

Supplementary Figure 4. Original Western blot scans of B7-H3 in 10 LuCap PDX series. LNCAP, positive control; HL-60s, negative control. Each LuCap PDX tumor is duplicated. GAPDH was used as the loading control.
