# Supplementary material for: Integrative molecular analyses define correlates of high B7-H3 expression in metastatic castrate-resistant prostate cancer
Source: NPJ Precis Oncol. 2022 Nov 2;6:80. doi: 10.1038/s41698-022-00323-2 (PMC9630314; doi:10.1038/s41698-022-00323-2)
Supplement: Supplementary file 2 — REPORTING SUMMARY [file 41698_2022_323_MOESM2_ESM.pdf]

nature portfolio

Corresponding author(s): Justin Hwang
NPJPRECISIONONCOLOGY-02303R1

Last updated by author(s): Oct 10, 2022

Reporting Summary

Nature Portfolio wishes to improve the reproducibility of the work that we publish. This form provides structure for consistency and transparency in reporting. For further information on Nature Portfolio policies, see our Editorial Policies and the Editorial Policy Checklist.

Statistics

For all statistical analyses, confirm that the following items are present in the figure legend, table legend, main text, or Methods section.

n/a	Confirmed	
☐	☒	The exact sample size (n) for each experimental group/condition, given as a discrete number and unit of measurement
☐	☒	A statement on whether measurements were taken from distinct samples or whether the same sample was measured repeatedly
☐	☒	The statistical test(s) used AND whether they are one- or two-sided<br>*Only common tests should be described solely by name; describe more complex techniques in the Methods section.*
☐	☒	A description of all covariates tested
☐	☒	A description of any assumptions or corrections, such as tests of normality and adjustment for multiple comparisons
☐	☒	A full description of the statistical parameters including central tendency (e.g. means) or other basic estimates (e.g. regression coefficient) AND variation (e.g. standard deviation) or associated estimates of uncertainty (e.g. confidence intervals)
☐	☒	For null hypothesis testing, the test statistic (e.g. F, t, r) with confidence intervals, effect sizes, degrees of freedom and P value noted<br>*Give P values as exact values whenever suitable.*
☒	☐	For Bayesian analysis, information on the choice of priors and Markov chain Monte Carlo settings
☒	☐	For hierarchical and complex designs, identification of the appropriate level for tests and full reporting of outcomes
☐	☒	Estimates of effect sizes (e.g. Cohen's d, Pearson's r), indicating how they were calculated

Our web collection on statistics for biologists contains articles on many of the points above.

Software and code

Policy information about availability of computer code

Data collection	Datasets derived from public resources and these resources are provided within the article and below.<br>1. The MSKCC 201019 data are deposited in at NCBI GEO under accession GSE21032. The analyzed data can also be accessed and explored through the MSKCC Prostate Cancer Genomics Data Portal:<br>http://cbio.mskcc.org/prostate-portal/&#x201D. doi:10.1016/j.ccr.2010.05.026 (2010).<br>2. The SU2C/PCF20 data are available in Dataset S1 and at www.cbiportal.org, and have been deposited in GitHub, https://github.com/cBioPortal/datahub/tree/master/public/prad_su2c_2019.doi:10.1073/pnas.1902651116 (2019)<br>3. For the SUWC dataset21 , the accession number for the raw sequencing data reported in the paper is dbGAP: phs001648.v1.p1. doi:10.1016/j.cell.2018.06.039 (2018).<br>4. The GTEx22 data is available through the GTEx portal (www.gtexportal.org). doi:10.3390/jpm5010022 (2015).<br>5. For the single-cell sequencing (scRNA-seq) dataset26 , scRNA-seq expression and clustering data generated in this study are available at https://singlecell.broadinstitute.org/single_cell/study/SCP1244/transcriptional-mediators-of-treatment-resistance-in-lethal-prostate-cancer. Raw sequence data generated in this study are being deposited in dbGaP (accession phs001988.v1.p1). doi:10.1038/s41591-021-01244-6 (2021).<br>6. The proteomics data of the 369 cell lines used in Fig S1a was obtained from DepMap Portal using the Data Explorer feature. https://depmap.org/portal/interactive/.
Data analysis	The basic machine learning operation source code, input datasets, and output matrices are provided in the following link:<br>git@github.com:bergo015/GeneNetworkingB7H3.git

For manuscripts utilizing custom algorithms or software that are central to the research but not yet described in published literature, software must be made available to editors and reviewers. We strongly encourage code deposition in a community repository (e.g. GitHub). See the Nature Portfolio guidelines for submitting code & software for further information.

Data

Policy information about availability of data

All manuscripts must include a data availability statement. This statement should provide the following information, where applicable:
- Accession codes, unique identifiers, or web links for publicly available datasets
- A description of any restrictions on data availability
- For clinical datasets or third party data, please ensure that the statement adheres to our policy

Datasets derived from public resources and these resources are provided within the article. The basic machine learning operation source code, input datasets, and output matrices are provided in the following link: git@github.com:bergo015/GeneNetworkingB7H3.git

Human research participants

Policy information about studies involving human research participants and Sex and Gender in Research.

Reporting on sex and gender	n/a
Population characteristics	n/a
Recruitment	n/a
Ethics oversight	n/a

Note that full information on the approval of the study protocol must also be provided in the manuscript.

Field-specific reporting

Please select the one below that is the best fit for your research. If you are not sure, read the appropriate sections before making your selection.

☒ Life sciences ☐ Behavioural & social sciences ☐ Ecological, evolutionary & environmental sciences

For a reference copy of the document with all sections, see nature.com/documents/nr-reporting-summary-flat.pdf

Life sciences study design

All studies must disclose on these points even when the disclosure is negative.

Sample size	No prospective collection was made. We used the datasets from open sources. Statistical outcomes were previously determined in these public studies to interpret genomic features of such tumors.<br>1. MSKCC 2010 (n = 131, primary PC; n = 19, CRPC). doi:10.1016/j.ccr.2010.05.026 (2010).<br>2. SU2C/PCF (n = 208, mCRPC). doi:10.1073/pnas.1902651116 (2019).<br>3. SUWC (n = 101, mCRPC). doi:10.1016/j.cell.2018.06.039 (2018).<br>4. GTEx (n = 245, benign prostate tissue). doi:10.3390/jpm5010022 (2015).<br>5. He et al. Single-cell sequencing dataset. Paired cell samples from one patient are included. Cell number = 112 before enzalutamide treatment and Cell number = 83 after enzalutamide treatment. doi:10.1038/s41591-021-01244-6 (2021).<br>6. Public RNA (22Q2) and Proteomics data of 369 cancer cell lines from over 35 lineages through Data Explorer tool from the DepMap Portal (https://depmap.org/portal/interactive/).<br>PC, prostate cancer; mCRPC, metastatic castration-resistant prostate cancer
Data exclusions	No exclusions, except in He et al, we sought to only use the paired enzalutamide treated samples.
Replication	The difference cohorts were used to cross compare key findings. The results were consistent.
Randomization	This is not relevant to our study. The majority of bulk sequencing samples were all included based on individuals with prostate cancer.
Blinding	This is not applicable. All treatments within the cohorts were considered in the analyses.

Reporting for specific materials, systems and methods

We require information from authors about some types of materials, experimental systems and methods used in many studies. Here, indicate whether each material, system or method listed is relevant to your study. If you are not sure if a list item applies to your research, read the appropriate section before selecting a response.

Materials & experimental systems

n/a	Involved in the study	
☐	☒	Antibodies
☐	☒	Eukaryotic cell lines
☒	☐	Palaeontology and archaeology
☒	☐	Animals and other organisms
☒	☐	Clinical data
☒	☐	Dual use research of concern

Methods

n/a	Involved in the study	
☒	☐	ChIP-seq
☒	☐	Flow cytometry
☒	☐	MRI-based neuroimaging

Antibodies

Antibodies used	Primary Antibodies used:<br>Invitrogen B7-H3 Monoclonal [6A1] Cat # MAS-15693 anti-mouse 1:500<br>R&D Systems B7-H3, anti-goat (1:500) Cat# AF1027<br>Bethyl B7-H3 [CD276], anti-rabbit (1:500) Cat # A700-026<br>GAPDH (6C5) anti-mouse (1:1000) cat# sc-32233<br><br>Secondary Antibody used:<br>IRDye 800 CW Goat-anti Mouse (1:2000) Licor cat# 925-32210<br>IRDye 800 CW Goat-anti Rabbit (1:2000) Licor cat# 925-32211<br>HRP-Conjugated Mouse-anti Goat (1:2000) Santa Cruz SC2354
Validation	We validated the antibodies using negative (HL-60s) and positive controls (LNCaP) cell lines.

Eukaryotic cell lines

Policy information about cell lines and Sex and Gender in Research

Cell line source(s)	Acquired LNCAP, HL60s from ATCC
Authentication	Yearly STR from ATCC
Mycoplasma contamination	Quarterly tests for Mycoplasma (more like every 6 mos) using MycoAlert (https://www.promega.com/resources/pubhub/applications-notes/detecting-mycoplasma-using-the-mycoalert-kit-on-the-glomax-2020/)
Commonly misidentified lines<br>(See ICLAC register)	Not on the list
